# Burst Packet Loss Concealment Using Multiple Codebooks and Comfort Noise for CELP-Type Speech Coders in Wireless Sensor Networks

**DOI:** 10.3390/s110505323

**Published:** 2011-05-17

**Authors:** Nam In Park, Hong Kook Kim, Min A Jung, Seong Ro Lee, Seung Ho Choi

**Affiliations:** 1 School of Information and Communications, Gwangju Institute of Science and Technology (GIST), Gwangju 500-712, Korea; E-Mail: naminpark@gist.ac.kr; 2 Department of Computer Engineering, Mokpo National University, Jeollanam-do 534-729, Korea; E-Mail: majung@mokpo.ac.kr; 3 School of Information Engineering, Mokpo National University, Jeollanam-do 534-729, Korea; E-Mail: srlee@mokpo.ac.kr; 4 Department of Electronic and Information Engineering, Seoul National University of Science and Technology, Seoul 139-743, Korea; E-Mail: shchoi@snut.ac.kr

**Keywords:** speech coding, G.729, wireless sensor networks, packet loss concealment, comfort noise, burst packet loss, voice onset

## Abstract

In this paper, a packet loss concealment (PLC) algorithm for CELP-type speech coders is proposed in order to improve the quality of decoded speech under burst packet loss conditions in a wireless sensor network. Conventional receiver-based PLC algorithms in the G.729 speech codec are usually based on speech correlation to reconstruct the decoded speech of lost frames by using parameter information obtained from the previous correctly received frames. However, this approach has difficulty in reconstructing voice onset signals since the parameters such as pitch, linear predictive coding coefficient, and adaptive/fixed codebooks of the previous frames are mostly related to silence frames. Thus, in order to reconstruct speech signals in the voice onset intervals, we propose a multiple codebook-based approach that includes a traditional adaptive codebook and a new random codebook composed of comfort noise. The proposed PLC algorithm is designed as a PLC algorithm for G.729 and its performance is then compared with that of the PLC algorithm currently employed in G.729 via a perceptual evaluation of speech quality, a waveform comparison, and a preference test under different random and burst packet loss conditions. It is shown from the experiments that the proposed PLC algorithm provides significantly better speech quality than the PLC algorithm employed in G.729 under all the test conditions.

## 1. Introduction

**Figure 1.**
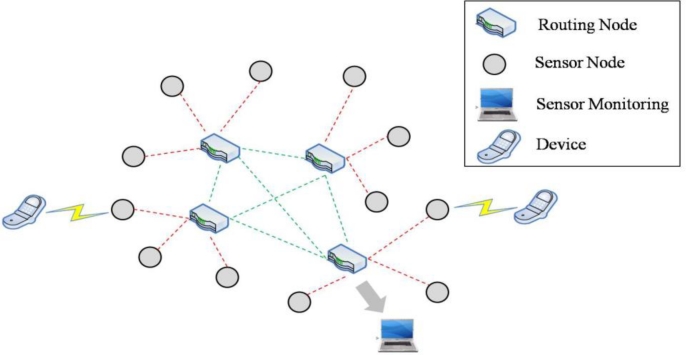
Structure of speech communications over WSNs.

There have been rapid developments in the wireless sensor networks (WSNs) field due to recent advances in related devices, such as new ultra low-power microcontrollers and short-rage transceivers. WSN technology is currently used in a wide range of applications including environmental monitoring, human tracking, biomedical research, military surveillance, and multimedia transmission [1,2]. As shown in Figure 1, we focus on speech data transmission suitable for speech communication over WSNs where each router node is connected by wireless local area network (WLAN) links and real-time transport protocol/user datagram protocols (RTP/UDPs) [3].

However, the unreliable transmission channels of wireless local area network (LAN) links and real-time transport protocol/user datagram protocols (RTP/UDPs) used in wireless sensor networks can cause significant packet losses or high latency in voice applications, as they have yet to be properly integrated into wireless senor network operations. Specifically, due to the nature of RTP/UDP transmissions in wireless sensor network environments, the packet loss rate becomes higher as the network becomes congested. In addition, depending on the network resources, the possibility of burst packet losses also increases, potentially resulting in severe degradation of reconstructed speech quality [4]. Since packet losses can occur in both wireless and wireline links, packet loss concealment (PLC) can become important in these networks.

Code-excited linear prediction (CELP) based speech coders are known to be sensitive to both bit errors and packet losses [5]. To reduce the quality degradation caused by packet losses, speech decoders should include a PLC algorithm. The packet loss concealment algorithms can be classified into the sender-based and receiver-based algorithms, depending on where the concealment algorithm works. The sender-based algorithms, for example forward error correction (FEC), require additional bits used for being processed in the decoder when frame losses occur [6]. On the other hand, the receiver-based algorithms, including repetition based concealment [7] and interpolative concealment [8], have advantages over the sender-based algorithms since they do not need any additional bits.

In this paper, a receiver-based PLC algorithm for CELP-type speech coders is proposed as a means of improving the quality of decoded speech under burst packet losses, especially when the packet loss occurs during voice onset intervals. The proposed PLC algorithm is based on a multiple codebook-based approach that includes a traditional adaptive codebook and a new random codebook composed of comfort noise to reconstruct decoded speech corresponding to the lost packets and the speech correlation-based PLC approach. Typically, CELP-type speech coders decompose speech signals into vocal track parameters and excitation signals. The former are reconstructed by repeating the parameters of the previous correctly received speech frame, while the latter are reconstructed by combining voiced and random excitations. In other words, voice excitation is obtained from the adaptive codebook excitation scaled by a voicing probability, whereas random excitation is generated by permuting the previous decoded excitation in order to compensate for an undesirable amplitude mismatch under burst packet loss conditions. However, this approach has difficulty in accurately reconstructing voice onset signals since parameters such as pitch period, linear predictive coding (LPC) coefficients, and adaptive/fixed codebooks of the previous frames are mostly related to silence frames [9]. The proposed PLC algorithm mitigates this problem by using a multiple codebook having comfort noise on the speech correlation-based PLC. The performance of the proposed PLC algorithm is then evaluated by implementing it on the G.729 speech decoder and comparing it to that of the PLC algorithm already employed in the G.729 speech decoder.

The remainder of this paper is organized as follows. Following this introduction, Section 2 describes a conventional PLC algorithm that is employed in the G.729 decoder [10]. After that, Section 3 describes the proposed PLC algorithm and discusses implemental issues. Section 4 then demonstrates the performance of the proposed PLC algorithm, and this paper is concluded in Section 5.

## 2. Conventional PLC Algorithm

**Figure 2.**
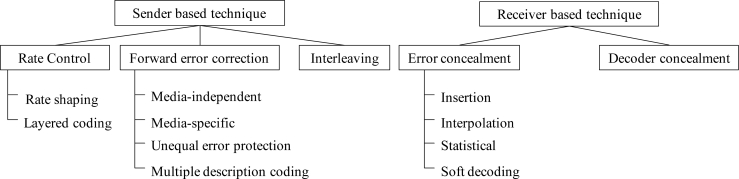
Classifications of packet loss concealment algorithms for speech packet transmission.

Figure 2 shows the classification of PLC algorithms, where each packet loss concealment algorithm can be classified as either a sender-based or a receiver-based algorithm, depending on the place where the PLC algorithm works [11–13]. As shown in the figure, rate shaping of sender-based algorithms is an active method of optimizing network resources and an attempt to adjust the rate of speech encoding according to current network conditions. Forward error correction (FEC) is a method by which the encoder sends extra information to help the decoder recover from packet losses. For example, media-independent channel coding is realized by using parity codes, cyclic redundancy codes, and Reed-Solomon codes, which enables the decoder to accurately repair lost packets without knowing the type of content. However, it entails additional delays and bandwidth. Another kind of media-specific FEC that attempts to make the decoder robust to bit error is unequal error protection (UEP), which protects only a part of the bits in each packet. Multiple description coding (MDC) is an alternative to FEC for reducing the effects of packet loss by splitting the bitstream into multiple streams or paths, though this technique consumes a wider bandwidth. The interleaving technique aims at distributing the effects of the lost packets such that the overall packet loss effects are reduced.

On the other hand, in the case of the receiver-based algorithms, the insertion-based error concealment (EC) techniques replace lost frames with silence, noise, or estimated values. Assuming that a future good packet will be available in the playout buffer just after a series of lost packets, interpolation-based EC techniques can be applied. The interpolation-based EC algorithm has the potential to reconstruct a lost frame by applying a linear or polynomial interpolation technique between the parameters of the first and last correct speech frames, before and after the burst packet loss. In general, the parameters of a lost frame are estimated by extrapolating those of a previous good frame. This approach works well for speech communication, where delay is an essential issue as no time should be lost waiting for future good frames at the decoder. Therefore, we focus on the extrapolating-based PLC technique which is performed only at the receiver.

In particular, the PLC algorithm already employed in G.729, which is here referred to as G.729-PLC, reconstructs speech signals of the current frame based on previously received speech parameters [7]. In other words, the algorithm replaces the missing excitation with an equivalent characteristic from a previously received frame, though this excitation energy tends to gradually decay. In addition, it uses a voicing classifier based on a long-term prediction gain. During the error concealment process, a 10 ms frame is declared as voiced if at least a 5 ms subframe of the frame has a long-term prediction gain of more than 3 dB; otherwise, the frame is declared as unvoiced. In this case, the lost frame inherits its class from the previous speech frame. The synthesis filter in the lost frame uses the linear predictive coding (LPC) coefficients of the last good frame, and the gains of the adaptive and fixed codebooks are attenuated by a constant factor, in which the pitch period of the lost frame uses the integer part of the pitch period from the previous frame. To avoid repeating the same periodicity, the pitch period is increased by one for each subsequent subframe.

## 3. Proposed PLC Algorithm

**Figure 3.**
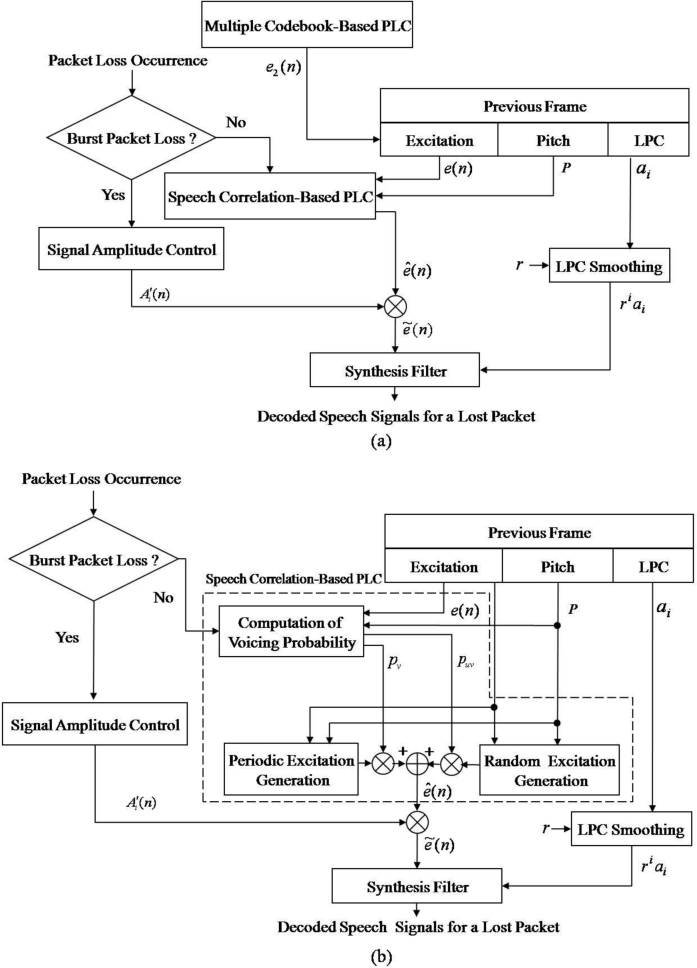
Overviews of **(a)** the proposed PLC algorithm and **(b)** the speech correlation-based PLC algorithm.

Contrary to G.729-PLC, the proposed PLC algorithm consists of two blocks: a speech correlation-based PLC (SC-PLC) block and a multiple codebook-based PLC (MC-PLC) block. The former includes voicing probability estimation, periodic/random excitation generation, and speech amplitude control; the latter incorporates comfort noise to construct multiple codebooks for reconstructing voice onset signals. Figure 3(a) shows an overview of the proposed PLC algorithm.

First, the multiple codebook, *e*_2_(*n*), is updated every frame regardless of packet loss. If the current frame is declared as a lost frame, LPC coefficients of the previous good frame are scaled down to smooth the spectral envelope. Next, a new excitation signal, *ê*(*n*), is estimated using the SC-PLC block, and then an updated multiple codebook is used to obtain *ẽ*(*n*) . Note that if consecutive frame losses occur, the signal amplitude estimate, $A_{i}^{\prime }(n)$, for the lost frame is obtained prior to the excitation estimation described above. Finally, decoded speech corresponding to the lost frame is obtained by filtering the estimated new excitation by using the smoothed LPC coefficients.

### 3.1. Speech Correlation-Based PLC

#### 3.1.1. Generation of Periodic and Random Excitations Using Voicing and Unvoicing Probabilities

Figure 3(b) also shows an overview of the SC-PLC block. This block attempts to estimate a new excitation signal, *ê*(*n*), for a lost frame by combining the periodic excitation obtained from the estimated voicing probability with the random excitation, where the random excitation is obtained by permuting the previously decoded excitation signal. Note here that the updated multiple codebook is used to generate the periodic and random excitations, which will be further explained in Section 3.2.

**Figure 4.**
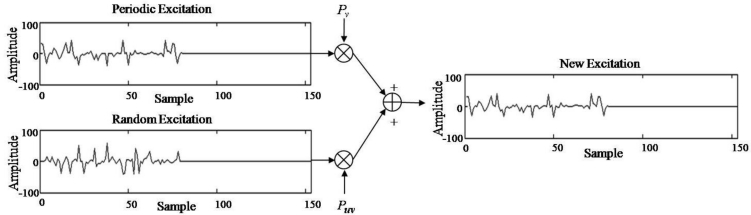
Example of generating excitation signals using the speech correlation block.

The SC-PLC algorithm generates the excitation of a lost frame by a weighted sum of the voiced and unvoiced excitations, which in turn is based on the pitch and the excitation of the previous frame, as shown in Figure 4. In particular, voiced excitation is first generated from an adaptive codebook by repeating the excitation of the previous frame during the pitch period, referred to as periodic excitation in this paper. That is, *e_p_* (*n*) is given by:

(1) $$e_{p}\;(n)\;=\;e(n-P)$$

where *e*(*n*) and *e_p_*(*n*) are the excitation of the previous frame and the periodic excitation, respectively, and *P* is the pitch period estimate of the current frame. Next, to generate unvoiced excitation, referred to as random excitation, temporal excitation is produced based on a random permutation of the excitation of the previous frame. That is, the temporal excitation, *e_t_*(*n*), is obtained by:

(2) $$e_{\text{t}}\;(n)\;=\;P_{\pi }\;(e(n))$$

where *P_π_* is the permutation matrix, and *n* is generated by a random sequence in the range of *P*. An excitation sample is then selected randomly from within a selection range having the same length as the pitch period. To select the next excitation sample, *P* is increased by one to prevent the same excitation sample from being selected.

In addition, assuming that the fixed codebook contributes somewhat to the periodicity of the speech signal as an adaptive codebook [14,15], we can compute the maximum cross-correlation between the periodic and temporal excitation as:

(3) $$m^{*}\;=\;\underset{0\leq m\leq N-1}{\text{arg}\;\text{max}}\;\frac{{\left(\sum_{i=0}^{N-1}e_{p}\;(n)\;\cdot \;e_{t}\;(n-m)\right)}^{2}}{\sum_{i=0}^{N-1}e_{\text{t}}^{2}\;(n-m)}$$

where *N* is the frame size, which is set to 80 for G.729. The best random excitation that contributes to the speech signal periodicity is then defined as:

(4) $$e_{r}\;(n)\;=\;e_{t}\;(n-m^{*})$$

where *e_r_*(*n*) is the random excitation. As shown in Figure 3, to recover the lost frame, we can obtain the reconstructed excitation by a weighted sum of the periodic and random excitation, such as:

(5) $$\hat{e}(n)\;=\;p_{v}e_{p}\;(n)\;+\;p_{uv}e_{r}\;(n)$$

where *ê*(*n*), *p_v_*, and *p_uv_* are the reconstructed excitation, voicing probability, and unvoicing probability, respectively. In Equation (5) *p_v_* and *p_uv_* are required in order to obtain the excitation. To this end, we first compute a correlation coefficient, *r*, between the excitation decoded in the previous frame and its delayed version, up to the estimated pitch period of the current frame *P* . In other words:

(6) $$r\;=\;\frac{\left|\sum_{n=0}^{N-1}e(n)e(n-P)\right|}{\sqrt{\sum_{n=0}^{N-1}e^{2}\;(n)}\;\sqrt{\sum_{n=0}^{N-1}e^{2}\;(n-P)}}.$$

Then, using the correlation coefficient, *p_v_* and *p_uv_* are estimated as:

(7) $$p_{v}\;=\;\{\begin{array}{ll} 1, & if\;r\;>\;0.33\\ \frac{r\;-\;0.03}{0.3}, & if\;0.03\;\leq \;r\;\leq \;0.33\\ 0, & otherwise \end{array}$$

and:

(8) $$p_{uv}\;=\;1\;-\;p_{v}.$$

The above probabilities are finally applied to Equation (5) in order to obtain the reconstructed excitation.

#### 3.1.2. Speech Amplitude Control Using Linear Regression

**Figure 5.**
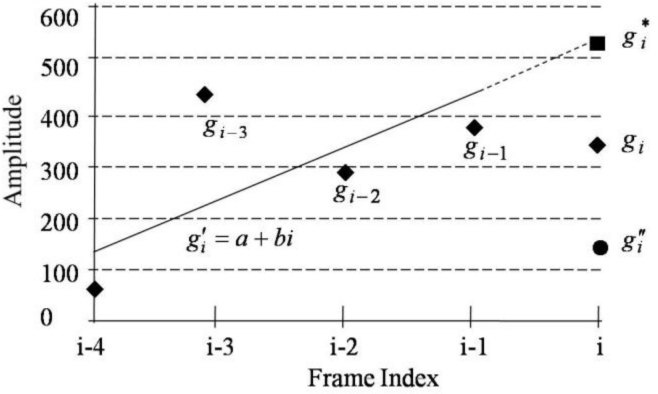
Amplitude prediction using a linear regression.

The SC-PLC algorithm described in Section 3.1.1 tends to reconstruct speech signals with relatively flat amplitudes, resulting in decoded speech of unnatural quality. To overcome this problem, we introduce a smoothing method to control the amplitude of decoded speech by using a linear regression technique. Figure 5 shows an example of the amplitude control. Assuming that *i* is the current frame and *g_i_* is the original speech amplitude, G.729-PLC estimates the amplitude $g_{i}^{\prime \prime }$ by attenuating the codebook gain, whereas SC-PLC estimates the amplitude $g_{i}^{*}$ using linear regression. In the figure, the amplitude obtained by linear regression provides a better estimate than the amplitude obtained by attenuating the codebook gain. Here, the linear regression is given by [16]:

(9) $$g_{i}^{\prime }\;=\;a\;+\;bi$$

where $g_{i}^{\prime }$ is the newly predicted current amplitude, *a* and *b* are coefficients for the first-order linear function, and *i* is the frame number. Assuming that measurement errors are normally distributed and that the past four amplitude values are available, we can find *a* and *b* such that the difference between the original speech amplitude and the speech amplitude estimated from Equation (9) is minimized. In other words, a^*^ and b^*^ are the optimized parameters with respect to *a* and *b*. Substituting these parameters into Equation (9), the amplitude estimate for the *i*-th frame is then denoted as:

(10) $$g_{i}^{*}\;=\;a^{*}\;+\;b^{*}i.$$

Next, to obtain the amplitude of a lost frame, the ratio of amplitude of the *i*-th current frame and that of the (*i*-1)-th frame is first defined as:

(11) $$\sigma_{i}\;=\;\frac{g_{i}^{*}}{g_{i-1}}$$

where *σ_i_* is the amplitude ratio of the *i*-th frame. Moreover, the number of consecutive lost frames is taken into account based on the observation that if consecutive frames losses occur, the speech amplitude also decreases. In this case, we define a scale factor, *s_i_*, as:

(12) $$s_{i}\;=\;\{\begin{array}{ll} 1.1, & if\;l_{i}\;=\;1,2\\ 1.0, & if\;l_{i}\;=\;3,4\\ 0.9, & if\;l_{i}\;=\;5,6\\ 0, & otherwise \end{array}$$

where *l_i_* is the number of consecutive lost frames until the *i*-th frame. Then, the estimated amplitude, $A_{i}^{\prime }$, can be determined using the equation:

(13) $$A_{i}^{\prime }\;=\;s_{i}\;\sigma_{i}.$$

Note that for continuous amplitude attenuation, $A_{i}^{\prime }$ is smoothed using the estimated amplitude of the (*i*-1)-th frame, $A_{i-1}^{\prime }$, as:

(14) $$A_{i}^{\prime }\;(n)\;=\;-\frac{A_{i-1}^{\prime }\;-\;A_{i}^{\prime }}{N}\;\cdot \;n\;+\;A_{i-1}^{\prime },\;n=0,\;\cdots ,N\;-\;1$$

where $A_{i}^{\prime }(n)$ is the smoothed amplitude of the *n*-th sample for the *i*-th frame. Finally, we multiply $A_{i}^{\prime }(n)$ to the excitation *ê*(*n*) to obtain the amplitude-adjusted excitation. That is, we have $\tilde{e}(n)\;=\;A_{i}^{'}(n)\hat{e}(n)$ and this value is subsequently applied to the synthesis filter.

### 3.2. Multiple Codebook-Based PLC

The SC-PLC block is unlikely to be able to accurately reconstruct voice onset signals. When the current frame is a voice onset, several previous frames could be silent or noise frames. Thus, if the current frame is lost, then coding parameters such as the pitch period, LPC coefficients, and excitation codebooks are not sufficient for reconstructing the current frame. To overcome this problem, we propose a multiple codebook-based PLC (MC-PLC) approach.

**Figure 6.**
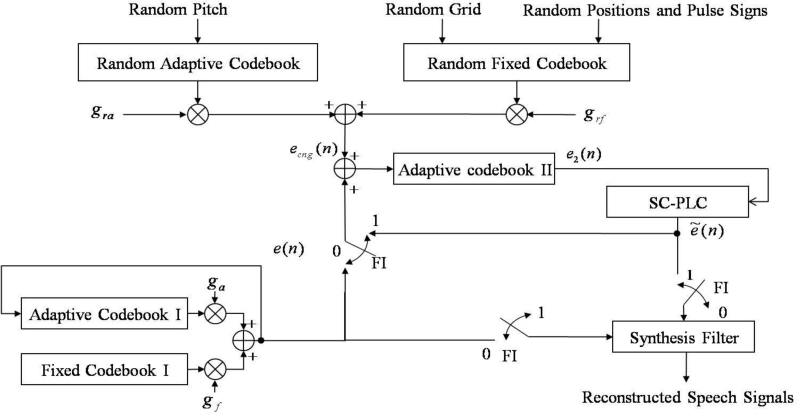
Structure of the proposed multiple codebook generation based on comfort noise, where FI is a frame erasure indicator.

Figure 6 shows the structure of the MC-PLC block. In this block, comfort noise is incorporated to make a secondary adaptive codebook, denoted as adaptive codebook II in the figure, to generate the excitation for a CELP-type coder. As shown in the figure, the adaptive codebook II excitation, *e*_2_(*n*), is used every frame without incurring frame loss. If there is no frame loss, *i.e.*, the frame indicator (FI) is set to 0, speech signals are reconstructed by filtering *e*(*n*). Simultaneously, the adaptive codebook II is updated as the sum of *e*(*n*) and *e_cng_*(*n*). Otherwise, the previous excitation of SC-PLC is substituted for *e*_2_(*n*). After applying *e*_2_(*n*) to SC-PLC, speech signals are reconstructed by filtering *ẽ*(*n*) . In this case, the adaptive codebook II is only updated by using the excitation sum of *ẽ*(*n*) by SC-PLC and *e_cng_*(*n*) by using the comfort noise. Here, *e_cng_*(*n*) is defined as:

(15) $$e_{cng}\;(n)\;=\;g_{ra}\;e_{ra}\;(n)\;+\;g_{rf}\;e_{rf}\;(n)$$

where *g_ra_* and *g_rf_* are the gains of the random adaptive codebook excitation, *e_ra_* (*n*), and the random fixed codebook excitation, *e_rf_* (*n*), respectively. In Equation (15)*e_cng_*(*n*) should be small compared to the excitation *e*(*n*) . In this paper, the squared sum of *e_cng_*(*n*) over a subframe is set to be below the squared sum of *e*(*n*), such that:

(16) $$\sum_{n=0}^{39}{\left(g_{ra}e_{ra}\;(n)\;+\;g_{rf}e_{rf}\;(n)\right)}^{2}\;=\;\alpha \sum_{m=0}^{39}{\left(e(n)\right)}^{2}\;\text{for}\;\alpha \;<\;1$$

where *α* is a scale factor and is adaptively set depending on the gain of the adaptive codebook I, *g_a_*, as shown in Figure 5. In other words, we have:

(17) $$\alpha \;=\;\{\begin{array}{ll} 0.48, & if\;g_{a}\;\geq \;0.6\\ 0.8g_{a}, & if\;0.12\;\leq \;g_{a}\;<\;0.6.\\ 0.108, & if\;g_{a}\;<\;0.12 \end{array}$$

Before solving Equation (16), we randomly choose *g_ra_* according to the rule that is already applied to generate the comfort noise in ITU-T Recommendation G.729 Annex B [10]. Finally, *g_rf_* is also obtained from Equation (16).

## 4. Performance Evaluation

To evaluate the performance of the proposed PLC algorithm, we replaced G.729-PLC [7] with the proposed PLC algorithm, and then measured the perceptual evaluation of speech quality (PESQ) scores according to ITU-T Recommendation P.862 [17]. For the PESQ test, 96 speech sentences, comprised of the utterances of 48 males and 48 females, were taken from the NTT-AT speech database [18] and processed by G.729 with the proposed PLC algorithm under different packet loss conditions. The performance was also compared to that using G.729-PLC. In this paper, we simulated two different packet loss conditions, which included random and burst packet losses in a wireless sensor network. During these simulations, packet loss rates of 3, 5, and 8% were generated by the Gilbert-Elliot model defined in ITU-T Recommendation G.191 [19–21]. Under the burst packet loss condition, the burstiness of the packet losses was set to 0.66; thus, the mean and maximum consecutive packet losses were measured at 1.5 and 3.7 frames, respectively.

**Figure 7.**
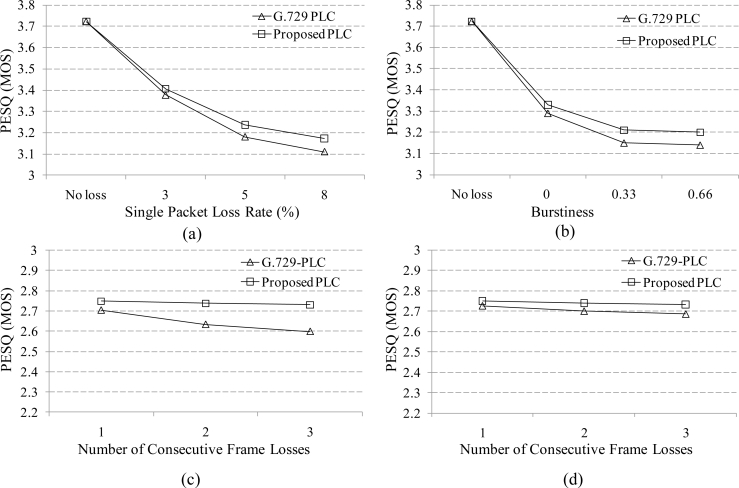
Comparison of PESQ scores of the proposed PLC and G.729-PLC under **(a)** single packet loss conditions and **(b)** burst packet loss conditions **(c)** of G.729-PLC and the proposed PLC according to different number of consecutive packet losses occurring during voice onset intervals **(d)** of G.729-PLC and the proposed PLC according to different number of consecutive packet losses occurring during non-voice onset intervals.

Figure 7(a,b) compares average PESQ scores when the proposed PLC and G.729-PLC were employed in G.729 under single packet loss and burst packet loss conditions whose burstiness was 0.66. In the figure, the proposed PLC algorithm had higher PESQ scores than the G.729-PLC algorithm for all conditions. In particular, the effectiveness of the proposed PLC algorithm was investigated when packet losses occurred during voice/non-voice onset intervals. In this end, we carried out a manual segmentation of voice/non-voice onset intervals. Figure 7(c,d) compares the PESQ scores for G.729-PLC and the proposed PLC under this simulated condition. It was shown from the figure that the proposed PLC provided the higher PESQ scores for any number of consecutive packet losses during the voice/ non-voice onset, respectively.

**Figure 8.**
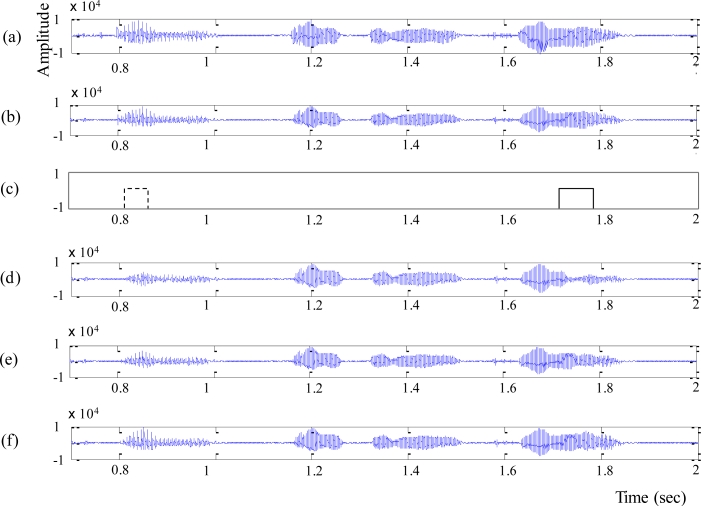
Waveform comparison: **(a)** original waveform, **(b)** decoded speech signal with no packet loss, and reconstructed speech signals using **(c)** packet error patterns, **(d)** G.729-PLC, **(e)** SC-PLC, and **(f)** the proposed PLC.

Next, we compared waveforms reconstructed by different PLC algorithms, which is shown in Figure 8. Figure 8(a,b) shows the original speech waveform and the decoded speech waveform with no loss of original signal, respectively. After applying the packet error pattern [expressed as a solid box in Figure 8(c)], it could be clearly seen that SC-PLC [Figure 8(e)] and the proposed PLC [Figure 8(f)] reconstructed the speech signals better than G.729-PLC [Figure 8(d)]. However, SC-PLC was unable to reconstruct the voice onset signal, as shown in the dotted box in Figure 8(c), which implied that the proposed PLC could provide better reconstruction of voice onset signals than SC-PLC.

Finally, in order to evaluate the subjective performance, we performed an A-B preference listening test, in which 10 speech sentences from five males and five females were processed by both G.729-PLC and the proposed PLC under random and burst packet loss conditions. Table 1 shows the A-B preference test results. As shown in the table, MC-PLC was significantly preferred to G.729-PLC for all the test conditions. On the average, the listeners preferred the proposed PLC more than three times than G.729-PLC.

## 5. Conclusions

In this paper, we have proposed a receiver-based packet loss concealment (PLC) algorithm for a CELP-type speech coder to improve the performance of speech quality when frame erasures or packet losses occurred in wireless sensor networks. The proposed PLC algorithm combined a speech correlation-based PLC (SC-PLC) with a multiple codebook-based (MC-PLC) approach. We subsequently evaluated the performance of the proposed PLC algorithm on G.729 under random and burst packet loss rates of 3, 5, and 8%, and then compared it with that of the PLC algorithm already employed in G.729. It was shown from PESQ tests, a waveform comparison, and A-B preference tests that the proposed PLC algorithm outperformed the PLC algorithm employed in G.729 under all the test conditions.

## Figures and Tables

**Table 1.** A-B preference score (%).

**Burstiness**	**Packet loss rate (%)**	**G.729 PLC**	**No difference**	**Proposed PLC**
γ = 0.0 (Random)	3 5 8	14.44 8.89 18.89	47.78 45.56 34.44	37.78 45.55 46.67
γ = 0.66 (Burst)	3 5 8	17.78 12.22 7.78	45.56 42.22 41.11	36.66 45.56 51.11
Average		13.33	42.78	43.89
